# Enzymatic Regulation and Biological Functions of Reactive Cysteine Persulfides and Polysulfides

**DOI:** 10.3390/biom10091245

**Published:** 2020-08-27

**Authors:** Tomohiro Sawa, Hozumi Motohashi, Hideshi Ihara, Takaaki Akaike

**Affiliations:** 1 Department of Microbiology, Graduate School of Medical Sciences, Kumamoto University, Kumamoto 860-8556, Japan; 2 Department of Gene Expression Regulation, Institute of Development, Aging and Cancer, Tohoku University, Miyagi 980-8575, Japan; hozumim@med.tohoku.ac.jp; 3 Department of Biological Science, Graduate School of Science, Osaka Prefecture University, Osaka 599-8531, Japan; ihara@b.s.osakafu-u.ac.jp; 4 Department of Environmental Medicine and Molecular Toxicology, Tohoku University Graduate School of Medicine, Miyagi 980-8575, Japan

**Keywords:** cysteine persulfide, antioxidant, anti-inflammatory effect, sulfur respiration, oxidative stress, cysteinyl-tRNA synthetase

## Abstract

Cysteine persulfide (CysSSH) and cysteine polysulfides (CysSS_n_H, *n* > 1) are cysteine derivatives that have sulfane sulfur atoms bound to cysteine thiol. Advances in analytical methods that detect and quantify persulfides and polysulfides have shown that CysSSH and related species such as glutathione persulfide occur physiologically and are prevalent in prokaryotes, eukaryotes, and mammals in vivo. The chemical properties and abundance of these compounds suggest a central role for reactive persulfides in cell-regulatory processes. CysSSH and related species have been suggested to act as powerful antioxidants and cellular protectants and may serve as redox signaling intermediates. It was recently shown that cysteinyl-tRNA synthetase (CARS) is a new cysteine persulfide synthase. In addition, we discovered that CARS is involved in protein polysulfidation that is coupled with translation. Mitochondrial activity in biogenesis and bioenergetics is supported and upregulated by CysSSH derived from mitochondrial CARS. In this review article, we discuss the mechanisms of the biosynthesis of CysSSH and related persulfide species, with a particular focus on the roles of CARS. We also review the antioxidative and anti-inflammatory actions of persulfides.

## 1. Introduction

**Figure 1:**
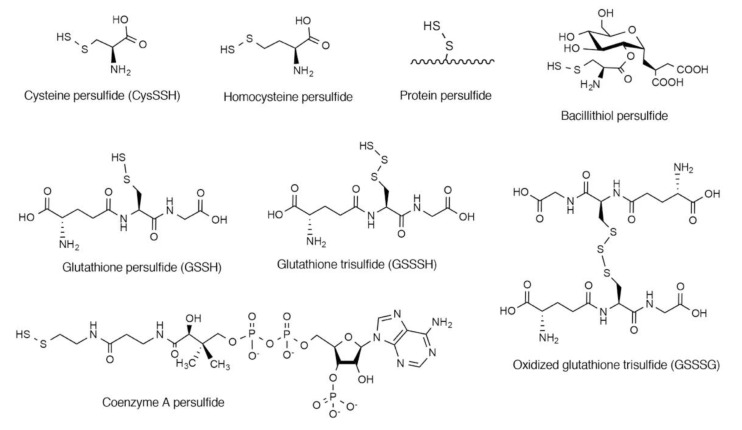
Chemical structures of persulfide species that have been identified in biological systems.

Cysteine persulfide (CysSSH) and cysteine polysulfides (CysSS_n_H, *n* > 1) are cysteine derivatives that have sulfane sulfur atoms that are bound to cysteine thiol [1,2,3,4]. Various forms of persulfides/polysulfides exist in both prokaryotes and eukaryotes as low-molecular-weight compounds, such as CysSSH, homocysteine persulfide, glutathione persulfide (GSSH), glutathione trisulfide (GSSSH), oxidized glutathione trisulfide (GSSSG), bacillithiol persulfide, and coenzyme A persulfide, and as CysSSHs in proteins (Figure 1) [5,6,7,8,9,10,11,12,13]. Persulfide species have been suggested to be involved in various biological processes. For example, it was reported that CysSSH serves as an important intermediate by donating its sulfane sulfur atoms during the biosynthesis of sulfur-containing biofactors such as iron–sulfur clusters, biotin, and lipoic acid [14,15]. Takahashi et al. demonstrated that CysSSH is involved in the regulation of tRNA methylthiolation and insulin secretion [16]. Calcium signaling mediated by Ca^2+^/calmodulin-dependent kinase I (CaMK I) can be regulated by protein sulfhydration at Cys179 [17,18]. CaMK I is activated by phosphorylation at Thr177, which is irreversibly inhibited by Cys177 persulfhydration [17,18]. Recent studies reported that CysSSH can participate in energy metabolism as it functions in sulfur respiration in mitochondria [3,4,6]. CysSSH produced by mitochondria-localized cysteinyl-tRNA synthetase is reduced in the presence of mitochondrial electron transfer chain activity to form hydrogen sulfide (H_2_S) [6]. H_2_S thus formed is likely followed by oxidation coupled with membrane potential generation [6]. CysSSH and related molecules can also act as strong nucleophiles and antioxidants and may have an important role in regulating oxidative stress and redox signaling in cells [1,5,6,7,19,20]. For instance, GSSH directly decomposed hydrogen peroxide (H_2_O_2_) in test tubes where parental GSH did not [5]. In addition, cell treatment with the GSSH donor, GSSSG, protected the cells from H_2_O_2_-induced cell death [7]. We recently demonstrated that CysSSH and related molecules have potent anti-inflammatory properties [13]. In this review article, we discuss the possible mechanisms of the biosynthesis of CysSSH and related persulfides and polysulfides. We also evaluate recent reports concerning the physiological functions of these compounds in view of the antioxidative and anti-inflammatory actions of CysSSH and related persulfides/polysulfides. Readers may also find other review articles on the roles of CysSSH in sulfur respiration of interest [3,4].

## 2. Endogenous Occurrence of CysSSH and Related Molecules

Recent advances in analytical methods that detect and quantify persulfides and polysulfides have shown that CysSSH and related species such as GSSH occur physiologically and are prevalent in prokaryotes, eukaryotes, and mammals in vivo. Liquid chromatography-tandem mass spectrometry (LC-MS/MS) with thiol-reactive reagents for stabilization of unstable persulfides and polysulfides is a powerful method for precise and sensitive quantification of persulfides and polysulfides. Iodacetamide [21] and monobromobimane [5] have been used as thiol-reactive reagents. However, detailed investigation revealed that polysulfur structures (-S-[S]n-S-, *n* > 1) decomposed during the reactions with those thiol-reactive reagents [22]. Hamid et al. have recently found that an iodacetamide derivative, possessing a hydroxyphenyl moiety (β-(4-hydroxyphenyl)ethyl iodacetamide; HPE-IAM) was a suitable persulfide/polysulfide reactive reagent with little decomposition of persulfides or polysulfides. They demonstrated that the hydroxyphenyl moiety can stabilize polysulfur structures [22].

By using LC-MS/MS methods, CysSSH and GSSH were detected in a variety of cell types including human lung adenocarcinoma A549 cells [5], neuroblastoma SH-SY5Y cells [5], cervical cancer HeLa cells [23], embryonic Kidney HEK293 cells [6], bronchial epithelial cells [8], rat glioma C6 cells [5], and mouse macrophage-like Raw264.7 cells [13]. Cellular levels of those persulfide and polysulfide species were influenced by culture conditions such as the overexpression of cysteine-metabolizing enzymes and the availability of sulfur-containing amino acids in the cultures, as well as the origins of the cells. Overexpression of cystathione γ-lyase (CSE), an enzyme that produces CysSSH from cystine (see later), resulted in a marked increase in CysSSH and GSSH in A549 cells [5]. On the other hand, gene knockout of cysteinyl-tRNA synthetases (CARSs), enzymes that produce CysSSH from cysteine (see later), led to significant reduction in cellular CysSSH and GSSH in HEK293 cells [6]. The inhibition of cystine uptake by sulfasalazine, an inhibitor of cystine transporter xCT, also resulted in a reduction in cellular CysSSH and GSSH in A549 cells [5]. LC-MS/MS-based analyses demonstrated the occurrence of CysSSH and related molecules in mice. Among mouse organs, the brain was found to contain the highest concentrations of GSSH compared to other organs such as the heart, liver, plasma, and lung. In the brain, GSSH levels were determined to be approximately 150 µM, which was 5% of total GSH contents [5]. It was found that the availability of dietary sulfur sources, including cystine and methionine, affected endogenous CysSSH and related molecules in vivo [5]. Cystine depletion and/or a reduction in methionine contents in the diet caused a reduction in CysSSH, GSSH, and GSSSH in mice [5].

The endogenous occurrence of CysSSH and GSSH has been reported in human tissues. Akaike and colleagues demonstrated the presence of various persulfide species including CysSSH, HCysSSH, GSSH, CysSSSSCys, CysSSSSSCys, and GSSSG in human plasma [5]. It is noteworthy that endogenous levels of persulfide species in humans were associated with certain diseases, such as chronic obstructive pulmonary disease (COPD). An inflammatory disease, COPD mainly affects small airways and lung parenchyma and leads to progressive airway obstruction [24]. Oxidative stress in the lungs has been suggested as the major etiological factor for COPD [24]. Ichinose and colleagues investigated endogenous levels of persulfide species in the lungs and found that levels of persulfide species, including CysSSH, GSSH, and GSSSH, were reduced in lung-resident cells and in epithelial lining fluid obtained from airways of patients with COPD [8]. They also observed that the levels of these persulfide species in lung cells were positively correlated with the extent of airflow limitation [8]. They further investigated the alteration of endogenous persulfide levels in patients with asthma-COPD overlap (ACO) [25]. ACO is defined as having the features of both asthma and COPD [26]. In general, patients with ACO are reported to have more frequent exacerbations, poorer quality of life, and rapid loss of lung function compared to patients with COPD alone. Total persulfides and polysulfides in patient’s sputum were measured by using the persulfide-reactive fluorescent probe SSP-4 [5]. Persulfide levels were significantly decreased in sputum from the patients with ACO compared with those from the healthy subjects and asthmatic patients [25]. In addition, persulfide levels were inversely correlated with values of 3-nitrotyrosine-immunopositive cells, a biomarker of oxidative stress [25]. Persulfide species have been shown to function as strong antioxidants to eliminate toxic oxidants [5]. These data thus suggest that a decrease in these persulfide species would be associated with a redox imbalance in the lungs of patients with COPD and/or with ACO.

Nakazawa and colleagues reported the endogenous occurrence of persulfide species in the aqueous and vitreous humour [7]. They measured persulfides and polysulfides by means of LC-MS/MS with HPE-IAM, and found that patients with diabetes mellitus (DM) had elevated levels of CysSSH, cystine, and GSSSG in the aqueous humour compared with healthy subjects. Similarly, patients with DM had increased levels of CysSSH, CysSH, and cystine in the vitreous humour compared with control subjects. In contrast, no significant difference in plasma levels of those persulfide species was observed between patients with DM and control subjects. Thus, various persulfide species appear to be present in the eye, and some persulfide species are up-regulated in the aqueous and vitreous humour in DM. Further study is warranted to clarify the roles of this up-regulation to compensate for oxidative stress in eyes with DM.

*Staphylococcus aureus*, a commensal pathogen of humans, is a major cause of nosocomial infections [27]. Giedroc and colleagues investigated the occurrence of persulfide species in this bacterium by using LC-MS/MS with monobromobimane derivatization [10]. They demonstrated the presence of persulfides of bacillithiol, cysteine, and coenzyme A in *S. aureus* [10]. Intracellular levels of these low-molecular-weight persulfides were affected by both genetic background and environmental factors [10]. Deficiency of the copper-sensing operon repressor (CsoR)-like sulfurtransferase repressor (CstR) gene, which is a transcriptional repressor of sulfide oxidation enzymes including CstA, CstB, and sulfide:quinone oxidoreductase (SQR), resulted in a reduction in persulfide species in *S. aureus* [10]. We discuss the roles of CstR in persulfide regulation in detail later in this review. Exposure of *S. aureus* to biologically relevant oxidants such as nitroxyl and peroxynitrite transiently increased the intracellular levels of low-molecular-weight persulfide species [10]. Takagi and Ohtsu reportedly detected persulfide species including CysSSH and GSSH in the Gram-negative bacterium *Escherichia coli* via LC-MS/MS with use of monobromobimane [28]. They identified those reactive species using mass spectrometry, although they reported no quantitative data [28].

Khan et al. reported that pathogenic bacterium *Salmonella enterica* serovar Typhimurium can endogenously produce CysSSH and related molecules dependent on the asymmetric sulfate reduction system [11]. Deletion of sulfate reductase genes, phs and asr, remarkably reduced bacterial persulfide and polysulfide levels [11]. Importantly, mutant bacteria lacking those genes were more susceptible to macrophage-mediated bacterial killing. Khan et al. demonstrated that bacterial surface proteins were modified by electrophilic nucleotide 8-nitroguanosine 3′,5′-cyclic monophosphate (8-nitro-cGMP) derived from macrophages, and such protein modifications triggered autophagy-mediated bacterial killing in macrophages [11]. Bacterial persulfides and polysulfides inactivated 8-nitro-cGMP so that it did not modify surface proteins and trigger autophagy [11]. Taken together, these observations suggest that persulfides and polysulfides play important roles in the protection against oxidative and electrophilic stresses, which will be discussed in more detail in Section 4 below.

## 3. Biosynthesis of CysSSH and Related Molecules

**Figure 2:**
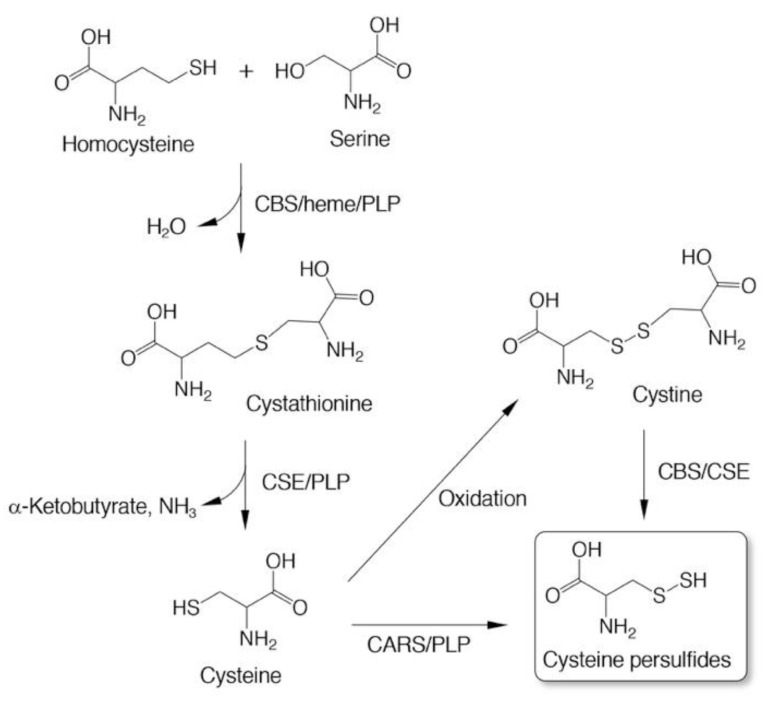
Enzymatic production of cysteine persulfides (CysSSHs). CBS, cystathionine β-synthase; CSE, cystathionine γ-lyase; CARS, cysteinyl-tRNA synthetase. Cofactors heme and pyridoxal phosphate (PLP) are also indicated.

Three enzymes—cystathionine β-synthase (CBS), CSE, and CARS—have been found to catalyze the formation of CysSSH as a product [3,5,6]. CBS and CSE are well-known as rate-limiting enzymes that are implicated in the transsulfuration pathway to form cysteine from homocysteine (Figure 2) [29,30]. In vitro experiments with recombinant enzymes showed that both CBS and CSE catalyzed the formation of CysSSH from cystine, an oxidized form of cysteine, that was used as a substrate (Figure 2) [5]. An analysis of products indicated that both CSE and CBS most likely go through an l-cystine C-S lyase-like reaction to form CysSSH [5]. Both enzymes, however, could not utilize cysteine for CysSSH formation [5].

**Figure 3:**
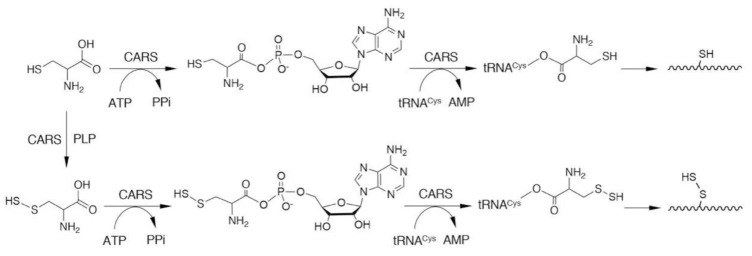
CARS-mediated incorporation of CysSSH into cysteinyl-tRNA and translation-coupled protein S-sulfhydration. PPi, pyrophosphate.

**Figure 4:**
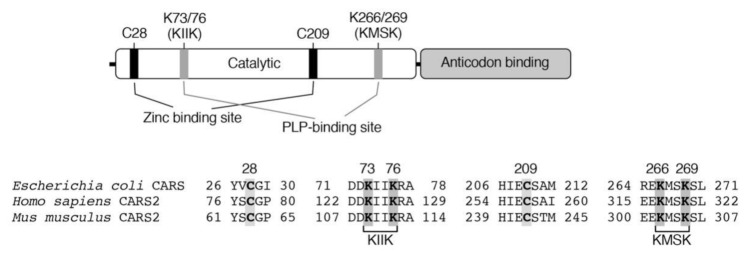
Domain structure and key amino acid residues involved in the aminoacylation and pyridoxal phosphate (PLP) binding of CARS.

CARSs are enzymes that catalyze cysteinyl-tRNA production by using a two-step mechanism, in which cysteine is first activated in the presence of ATP to form an enzyme-bound cysteinyl adenylate (Figure 3) [31,32,33]. In the second step, activated cysteine is transferred to the 2′-ribosyl OH group at the 3′-terminus of the cysteinyl-tRNA molecule (Figure 3). CARS contains one Zinc atom in the active site, that plays an important role for substrate cysteine thiolate binding [34]. The Zinc ion is kept to the active site through coordination of the side-chains of C28, C209, H234, and E238 of the enzyme, and hence, those amino acid residues play critical roles in the aminoacylation reaction catalyzed by CARS (Figure 4). The introduction of cysteine-to-aspartate mutations at C28D and C209D in CARS was found to result in a considerable reduction in protein synthesis in the cell-free PUREfrex system as well as in cultured cells [6].

Akaike and colleagues determined that not only bacterial (*Escherichia coli, E. coli*) but also mammalian (human and mouse) recombinant CARSs can catalyze the formation of CysSSH from cysteine that was used as a substrate (Figure 2 and Figure 3) [6]. This substrate specificity contrasts with those for CSE and CBS; CSE and CBS utilize only cystine (but not cysteine) as a substrate for CysSSH production [5]. Kinetic analysis confirmed a Michaelis constant *K*_m_ of 7.3 ± 0.9 µM and a *k*_cat_/*K*_m_ value of 1.4 ± 0.3 × 10^3^ M^–1^ s^–1^ for an *E. coli* CARS with the substrate cysteine [6]. The intracellular cysteine concentrations are reportedly in the range of 100–1000 µM in cells and major organs [5], which is much higher than the *K*_m_ of CARS. Biochemical and structural analyses revealed that CARSs use pyridoxal phosphate (PLP) as a cofactor for CysSSH formation [6]. In CARSs, lysine residues at K73, K76, K266, and K269 are well conserved (Figure 4). Mass spectrometry-based proteomic analysis of recombinant *E. coli*-CARS showed that PLP-bound lysine residues were determined to those sites [6]. Some of these lysine residues such as K266 and K269 were found to be PLP-bound even without exogenous PLP addition [6]. The addition of PLP to recombinant human CARS2 increased PLP-bound lysine residues in a dose-dependent manner. Importantly, enzyme reactions catalyzed by human recombinant CATS2 significantly enhanced the formation of CysSSH by more than 10 times in the presence of 10 µM PLP compared to those without the addition of PLP [6]. In addition, enzymes that had lysine-to-alanine mutations introduced at K73, K76, K266, and K269 showed markedly less activity in terms of CysSSH formation, whereas these mutant enzymes had almost intact protein synthesis activity [6].

In mammalian cells, two different CARSs exist—the cytosolic CARS1 and the mitochondrial CARS2 [35,36]. Studies with recombinant enzymes showed that both CARS1 and CARS2 possess strong CysSSH-producing activity [6]. The importance of CARS2 for producing persulfide and polysulfide species in vivo was also supported by the fact that endogenous low-molecular-weight persulfide levels in CARS2 heterozygous KO mice (Cars2^+/–^) were markedly reduced, by almost 50%, compared with the levels in wild-type mice [6].

Protein persulfides (i.e., protein S-sulfhydration or persulfidation; Figure 1) have been found in various proteins [5,6,37,38,39]. Accumulating evidence suggests that protein persulfidation may have an important role in regulating protein functions [17,18,40,41,42]. Previous studies reported that protein persulfides were formed as a result of the post-translational modification of thiols that were mediated by certain sulfur-donating molecules such as H_2_S [43,44,45]. A noteworthy discovery was that low-molecular-weight persulfide species such as GSSH can donate their sulfur atoms quite efficiently to acceptor protein thiols to form protein-bound CysSSH [5]. In addition to demonstrating post-translational modification, Akaike and colleagues showed, for the first time, that CARSs can catalyze the direct incorporation of CysSSH into nascent polypeptides during translation (Figure 3) [6]. In fact, CysSSH bound to cysteinyl-tRNA was discovered in in vitro reactions involving cysteine, recombinant CARS, cysteinyl-tRNA, and ATP [6]. An analysis of nascent polypeptides obtained from *E. coli* ribosomes showed an extensive formation of peptide persulfides after translation [6]. We thus suggest that the translation-coupled incorporation of CysSSH into proteins that are catalyzed by CARSs is an important mechanism for the maintenance of protein persulfides.

## 4. Antioxidative and Nucleophilic Properties of Persulfides/Polysulfides

As mentioned earlier, strong nucleophilicity and potent antioxidative activity are unusual features of CysSSH and cysteine polysulfide species [2,19,21]. We previously demonstrated that GSSH derived from the glutathione reductase-mediated reduction of oxidized glutathione trisulfide (GSSSG) very efficiently decomposed H_2_O_2_ [5]. Under the same conditions, parental reduced glutathione (GSH) or H_2_S failed to reduce H_2_O_2_ [5]. Li et al. reported that GSSH was 50-fold more reactive than was H_2_S toward H_2_O_2_ at physiological pH [46]. In cell systems, the overexpression of CSE enhanced GSSH levels and, more importantly, protected cells from H_2_O_2_-induced death [5]. Kunikata et al. also reported that the exogenous addition of GSSSG significantly suppressed cultured cell death induced by H_2_O_2_ exposure [7]. These data suggest that persulfide/polysulfide species may act as important antioxidants inside cells and protect cells from oxidative stress.

Everett and Wardman reported that persulfides can efficiently scavenge free radical species [47]. Persulfides are stronger acids than are thiols, so at physiological pH (at which many thiols occur predominantly in the protonated form), a significant proportion of hydropersulfide species exist as a deprotonated persulfide anion (RSS^–^) [19]. In fact, Li et al. recently reported that the p*K*_a_ of GSSH was 6.9, that is, two orders of magnitude smaller than that of GSH (p*K*_a_ = 8.9) [46]. RSS^–^, thus formed, readily reacted with radical species to form persulfide radicals (RSS•) [47]. RSS• are considerably less reactive compared to their thiyl radical counterparts, and hence, are probably less toxic if produced in cells. Persulfide/polysulfide species can also efficiently react with various electrophiles [5,48]. 8-Nitro-cGMP is an endogenously occurring weak electrophile and acts as a second messenger in redox signaling [49,50]. We found that persulfide/polysulfide species reacted with 8-nitro-cGMP to convert it to 8-mercapto-cGMP [5]. These findings demonstrate the unusual potency of persulfide/polysulfide species as strong reductants and nucleophiles, so that they manifest greater reactivity than thiols and can scavenge oxidants and cellular electrophiles, thereby serving as important antioxidant molecules.

## 5. *N*-Acetyl-l-Cysteine (NAC) Polysulfides as Useful Chemical Tools to Investigate Biological Functions of Reactive Sulfur Species

Polysulfur donors that can raise persulfide/polysulfide levels in cells by donating sulfur atoms to endogenous acceptor thiols become important chemical tools to help understand the physiological and pathological roles of biological reactive sulfur species. Powell et al. reported that NAC conjugated to pinacolate boronate (Bpin) through disulfide bonding can act as a prodrug for a persulfide donor (BDP-NAC) [51]. BDP-NAC can release free NAC hydropersulfide after exposure to H_2_O_2_ [51]. Powell et al. demonstrated that treatment with BDP-NAC protected cells from death caused by H_2_O_2_ exposure, possibly by producing the antioxidant NAC hydropersulfide in cells [51]. Zheng et al. developed a persulfide prodrug that can produce a hydroxymethyl persulfide by means of esterase-dependent activation [52]. They also reported that such a persulfide-generating prodrug had strong cardioprotective effects, with a bell-shaped therapeutic profile, in a murine model of myocardial ischemia-reperfusion injury [52]. Kang et al. reported another type of persulfide precursor that produces hydropersulfides via pH- or F^–^-mediated desilylation of *O*-silyl mercaptan-based molecules that contain sulfur [53]. Chaudhuri et al. showed that photon-activated CysSSH donors were involved in the spatiotemporal control of persulfide generation [54].

**Figure 5:**
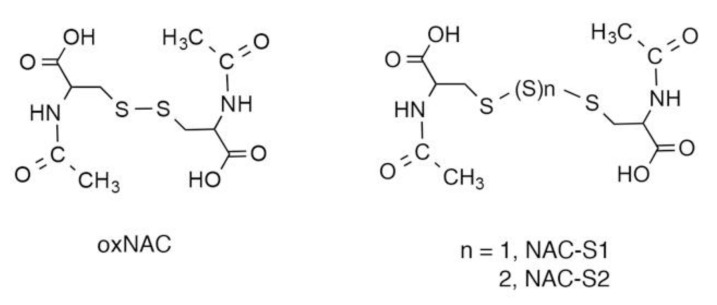
Chemical structures of NAC polysulfides. oxNAC is a control reagent without polysulfide-donating capability.

One unique characteristic of persulfides/polysulfides is that they can donate sulfane sulfur atoms to acceptor thiols by means of sulfur transfer reactions [2,19,23]. Related to this finding, we demonstrated that NAC polysulfides (Figure 5) quite efficiently increased intracellular levels of reactive sulfur species such as CysSSH and GSSH [2]. Mass spectrometric analyses clearly indicated that NAC polysulfides were rapidly incorporated into cells and donated their sulfur atoms to endogenous acceptor thiols such as cysteine and GSH [13]. NAC polysulfides may have certain advantages as persulfide/polysulfide donors. First, these polysulfides are stable during storage, even in aqueous media [13], which should help researchers avoid the inconvenience of preparing fresh solutions just before each experiment. Second, researchers can easily label NAC polysulfides with ^34^S at sulfane sulfur moieties by using ^34^S-sulfide as a starting material. The transfer of sulfur from ^34^S-labeled NAC polysulfides and acceptor thiols in cells can therefore be analyzed by monitoring isotope-targeted sulfur metabolomics in complex biological milieu [13]. In addition, the metabolism of persulfides/polysulfides, especially with regard to their sulfane sulfur atoms, may be studied by analyzing sulfur metabolites labeled with ^34^S. Third, we expect that NAC polysulfide-derived decomposition metabolites, including NAC, would be biologically inert. Therefore, we can anticipate that NAC polysulfides may be used as prototype therapeutic drugs if they have favorable effects.

## 6. Anti-inflammatory Actions of NAC Polysulfides

**Figure 6:**
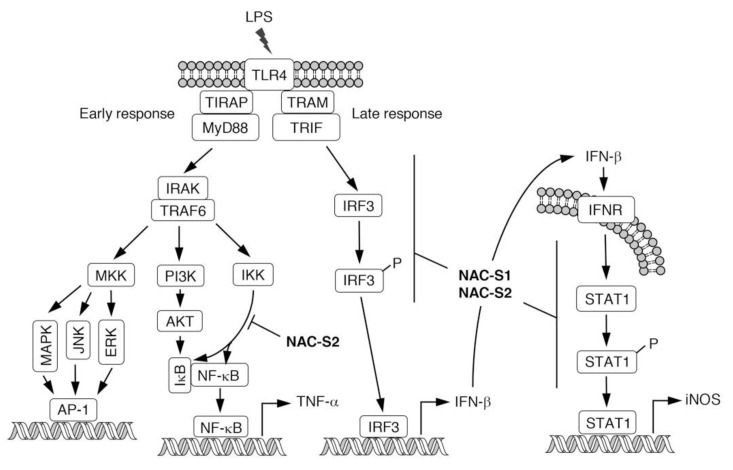
Schematic representation of LPS-TLR4-mediated inflammatory responses. NAC polysulfides can inhibit signal transduction at the points indicated. LPS, lipopolysaccharide; TLR4, toll-like receptor 4; MKK, MAP kinase kinase; MAPK, mitogen-activated protein kinase; JNK, c-Jun NH_2_-terminal kinase; ERK, extracellular signal-regulated kinase; PI3K, phosphatidylinositol-3 kinase; IKK, IκB kinase; AKT, protein kinase B; IRF3, interferon regulatory factor 3; INFR, interferon receptor; STAT1, signal transducers and activators of transcription 1; iNOS, inducible nitric oxide synthase.

Although modulatory effects of endogenous H_2_S derived from CSE on immune responses have been reported [55,56], the molecular mechanisms of how those enzymes regulate immune responses remain unclear. We previously demonstrated, by using polysulfur donor NAC polysulfides, that CysSSH and cysteine polysulfide species negatively regulated innate immune responses augmented by lipopolysaccharide (LPS) in vitro and in vivo [13]. Macrophages are immunologically stimulated by LPS treatment to produce proinflammatory cytokines such as tumor necrosis factor-α (TNF-α) and interferon-β (IFN-β) (Figure 6). NAC polysulfide treatment was demonstrated to markedly inhibit the production of both TNF-α and IFN-β by cells of the mouse macrophage cell line Raw264.7 [13]. Several phosphorylation-transcription factor signals are activated in response to LPS to produce TNF-α, via pathways such as Iκ kinase (IKK)- and protein kinase B (AKT)-nuclear factor-κB (NF-κB), and mitogen-activated protein kinase (MAPK)-/c-Jun N-terminal kinase (JNK)-/extracellular signal-regulated protein kinase (ERK)-AP-1 [57,58]. We found that treatment with NAC polysulfides had different effects on the phosphorylation network downstream of LPS-toll-like receptor 4 (TLR4) signaling, and inhibition of the IKK/NF-κB axis may contribute to the suppression of TNF-α production caused by NAC polysulfide treatment [13] (Figure 6). In innate immune responses, different TLRs recognize various ligands such as zymosan A (by TLR2) and viral RNA duplex (by TLR3) [59,60]. NAC polysulfides were suggested to inhibit not only TLR4 but also TLR2 and TLR3 by suppressing IKK/NF-κB signaling [13]. In addition to inhibiting proinflammatory cytokine production, NAC polysulfides strongly suppressed cytokine-mediated inflammatory responses such as the expression of inducible nitric oxide synthase (iNOS). IFN-β released extracellularly can activate the signal transducer and activator of transcription 1 (STAT1) signaling, which leads to production of the inflammatory mediator nitric oxide via expression of iNOS, in an autocrine or paracrine manner (Figure 6). NAC polysulfides reportedly suppressed IFN-β-dependent inflammatory responses by inhibiting both IFN-β production and STAT1 phosphorylation [13] (Figure 6).

Macrophage activation in response to LPS is an important innate immune response that helps in the eradication of infecting Gram-negative bacteria. However, macrophages continuously exposed to LPS or exposed to large amounts of LPS result in the introduction of excess amounts of pro-inflammatory cytokines (i.e., a cytokine storm), which finally leads to lethal endotoxin shock [61,62]. Of endotoxin shock model mice that received LPS intraperitoneally, 80% of the animals died within 96 h [13]. NAC polysulfide treatment markedly improved the survival rate of the mice: only 10% of mice died 96 h after LPS administration [13]. These data suggest that NAC polysulfides have anti-inflammatory functions in vivo, possibly through the suppression of LPS-induced inflammatory responses.

Excessive and dysregulated activation of TLR4 signaling has been associated with the development of various inflammatory diseases, as well as endotoxin shock pathology [62,63,64]. A lot of compounds have been tested in animal models to evaluate their capacity to block TLR4-mediated cytokine production, with several compounds undergoing clinical trials [62,63,64]. As mentioned above, NAC polysulfides have potent anti-inflammatory effects, and hence, may become a new class of TLR4 antagonists that can be used to treat endotoxin shock. Quite recently, TLR4 was suggested to be a promising therapeutic target in drug abuse [65] and major depressive disorders [66], as well as amyotrophic lateral sclerosis [67]. The possible use of TLR4 antagonists to treat peripheral neuropathic pain was also reported [68]. Persulfide/polysulfide donors, solely or in combination with other TLR4 antagonists, warrant continued investigation as potential therapeutic options.

## 7. Conclusions

In summary, CysSSH and cysteine polysulfide species are found physiologically and are abundant in prokaryotes, eukaryotes, and mammals in vivo. The chemical properties and extensive biological formation of these species suggest a pivotal role for reactive persulfides in cell regulation. CARS can catalyze the production of CysSSH from cysteine as a substrate. CARS can also catalyze protein polysulfidation by direct incorporation of CysSSH into polypeptides. CysSSH and related species can behave as potent antioxidants and cellular protectants and may function as redox signaling intermediates. Other important physiological functions of CysSSH include mitochondrial biogenesis and bioenergetics via sulfur respiration regulated by CysSSH. Recent studies suggested that CysSSH and related species are intimately involved with the regulation of immune function and that pathological inflammatory responses can be improved by artificially increasing reactive sulfur species. Excessive inflammatory reactions occur not only in endotoxin shock but also in allergies and autoimmune diseases. Typical treatments of these disorders include steroid hormones and immunosuppressants, but these treatments have a variety of side effects. We expect to target intracellular sulfur regulation in the future so as to establish new anti-inflammatory therapeutic options.
